# Supplementary material for: Are carbon emissions trading and green financial instruments synergistic? -Comprehensive quantitative research based on content analysis
Source: PLoS One. 2024 Mar 7;19(3):e0298601. doi: 10.1371/journal.pone.0298601 (PMC10919601; doi:10.1371/journal.pone.0298601)
Supplement: S1 Data — (ZIP) [file pone.0298601.s001.zip › Data/地级市Z_F工作报告.zip!/║■▒▒/╩«╤▀╩╨/└┤╫╘╣1⁄2╓┌║┼í╛┬φ┐╦╩2╛▌═°í┐_2010.docx]

各位代表：

　　现在，我代表市人民政府向大会作工作报告，请予审议，并请各位政协委员和其他列席会议的同志提出意见。

　　一、2009年工作回顾

　　2009年是困难最大的一年，也是发展成效最好的一年。面对国际金融危机的影响和汽车产业开局受挫的严峻形势，在市委的坚强领导下，全市上下危中寻机，共克时艰，奋力拼搏，圆满完成了保增长、保民生、保稳定的任务，提前一年实现了“十一五”规划目标。预计全年实现生产总值550亿元，同比增长15.2%；地方一般预算收入25.8亿元，增长30.9%；全社会固定资产投资278.4亿元，增长50.1%；社会消费品零售总额254亿元，增长20.5%；外贸出口1.72亿美元，增长33.6%；实际利用外资5733万美元，增长13.6%；城镇居民人均可支配收入11376元，增长8%；农民人均纯收入3110元，增长9.5%；万元生产总值综合能耗下降4.8%；城镇登记失业率控制在4.5%以内；人口自然增长率控制在5.6‰；居民消费价格总指数累计上涨0.5%，市三届人大六次会议确定的主要目标任务全面和超额完成。

　　（一）全力破解发展困局，工业经济创历史新高。面对金融危机带来的一季度工业产值下滑16.5%的严峻局面，及时采取了一系列超常规应对举措，有力地促进了工业经济反跌复升，创造了月产、季产、年产“三个新高”。全市规模以上工业总产值达821亿元，增幅达25.4%，高于全省平均水平8.4个百分点，其中12月产值达到94.6亿元、第四季度产值达到266.4亿元，均刷新了历史记录。汽车工业实现产值589.8亿元，增长28.1%。地方工业强劲增长，实现产值433亿元，增长39.1%，其中50家“双亿工程”企业实现产值220亿元，净增60亿元，增长45.8%；地方工业占全部工业的比重达到52.7%。我市被授予“扶持中小企业创新发展成绩显著地方政府”称号。开发区发展势头迅猛，全市9个开发区工业产值增长35%以上，十堰经济开发区获得“中国最佳汽车产业示范开发区”称号。新的增长点不断显现，新增规模以上工业企业149家，达到771家；100个重点工业项目相继开工，76个项目投产。东风渝安20万辆扩能项目即将投产，三环6万辆和东风实业5万辆专用汽车扩能项目已开工；双星东风100万套全钢子午胎正式投产；世纪中远5万辆微型车扩能项目的生产线、涂装线建成；西部工业新区实现了“三通”、入驻项目13个。

　　（二）大力实施“一统三分”战略，农村经济快速发展。召开了地市合并以来的首次三级干部大会，制定和实施了“一统三分”战略，农村经济保持良好发展势头，实现了“两大突破”。全市第一产业产值突破百亿元大关，达111.9亿元，增长10%；农民人均纯收入突破3000元农村小康标准线。粮油丰产丰收，分别达到116.2万吨、10万吨，增长7.6%和18.6%。农业产业化加速推进，新增蔬菜、茶叶、烟叶、木瓜等特色基地20万亩、畜禽养殖小区120个、专业村34个、省级龙头企业7家、市级龙头企业10家、“三品”品牌（无公害食品、绿色食品、有机食品）20个、各类农民专业合作社119个；武当道茶被授予省第一文化名茶；房县黑木耳、丹江翘嘴鲌、武当蜜橘获得国家地理标志产品保护，马头山羊标准作为国家标准颁布。扶贫开发深入推进，贫困人口减少7万人，搬迁扶贫2755户、1.1万人。劳务经济平稳发展，转移农村劳动力66万人，实现劳务总收入42.3亿元。基础设施建设扎实推进，改造了11.2万亩低丘岗地，新修通村水泥路2500公里，建成农村五级站11个、候车亭180个、招呼站300个。完成了2座中型水库、21座小型水库的除险加固，解决了38.8万人饮水不安全问题。县域经济快速发展，五县一市实现生产总值220亿元，增长15%。

　　（三）突破性发展生态文化旅游业，第三产业持续繁荣。抢抓鄂西生态文化旅游圈建设机遇，一批景点和基础设施项目全力推进，武当山八大项目进展顺利，十堰至西安旅游客运专线开通，武神公路通车，武当山体育馆竣工，丹江口库区和武当山两个旅游码头正在建设；一批景点创优升级，武当山景区通过5A级旅游景区评审并被确定为国家地质公园，房县“野人谷”和“野人洞”、郧西五龙河旅游景区晋升为4A级景区。丹江口市通过中国优秀旅游城市验收，我市被评为全国最佳生态旅游城市和中国十佳休闲城市。全市接待国内外游客1123.7万人次，实现旅游收入62.3亿元，分别增长22%和41.8%，其中武当山接待游客150.2万人次，实现旅游收入8.3亿元，分别增长23.1%和48.2%。

　　商贸流通业快速发展。新建和改建农家店300家、农村示范超市106个、农资示范放心店112个，建立家电下乡销售网点493家；成功举办了2009中国·十堰汽车汽配订货会，供销合同金额达267.6亿元。工商、质监、食品药品、物价等部门强化市场监管，促进了市场繁荣。在旅游、商贸的有力带动下，全市第三产业实现增加值230亿元，增长13.4%。

　　（四）充分发挥服务发展职能，财政金融运行稳健。财税部门积极培植财源，强化税收征管，加强预算管理，保障重点支出，科学理财水平进一步提高。实现财政总收入55亿元，增长22.6%。金融机构加大产品创新力度，组织开展了“百亿基金进十堰”活动，成立了两家小额贷款公司，政信担保公司担保额度达到5.1亿元，放大倍数6.4倍，有力地支持了企业发展和项目建设。各项存款余额695亿元，比年初增加125亿元；各项贷款余额310亿元，比年初增加76亿元。[FS:PAGE]

　　（五）全力以赴争取政策和资金，重大项目建设取得突破性进展。以“项目建设年”为抓手，积极抢抓扩大内需的政策机遇，着力实施“重大项目百日攻坚”活动，资金争取和项目建设成效显著。争取国家对丹江口库区生态补偿资金3.81亿元；争取中央投资项目673个、资金13亿元。新开工项目784个，增加365个。重大交通项目建设取得突破，武当山机场场址选定；武汉至十堰动车组开通，襄渝铁路二线全线通车，十宜铁路正在争取国家立项，十三铁路完成预工可报告，郑渝铁路正在开展设计方案比选；福银高速十堰至西安段建成通车，十房、十白和谷竹三条高速公路开工建设，郧十高速公路完成初步设计；武白、郧十两条一级路建设快速推进；兴界路、郧漫路等干线公路改造完成；郧县汉江二桥、丹江口习均大桥加快建设，五条岭、大川和雷峰垭隧道已经贯通；汉江航道整治二期工程正在进行。水电项目建设进展顺利，潘口、小漩电站成功截流，孤山、龙背湾电站临建工程开工，新增水电装机8.3万千瓦。天然气十堰支干线工程即将动工，热电联产项目可研报告通过评审。

　　（六）深入推进“三城联创”，城镇化建设水平明显提升。启动了新一轮城市总体规划和土地利用总体规划修编工作。着力打破城市发展空间制约，规划、城投、国土等部门积极配合、主动服务，平整山地2万余亩；加快城市主干道建设，凯旋大道建成通车，山东路、建设大道一期工程、林荫大道二线建设正在推进，上海路一期工程、北京路南通道建设有序推进，柳林路、江苏路改造完成，六堰广场地下通道贯通，中心城区交通压力得到缓解。西部工业新区新建了捷达路、凯迪拉克大街，发展空间进一步拓展。

　　积极推进“三城联创”，城市功能进一步完善，生态建设和节能减排成效明显。城区60条内街小巷道路改造完成，新建3座人行天桥、10座公厕和垃圾中转站、8座街头游园和山体游园，高速客运站投入运营。赛武当通过国家级自然保护区评审，红岩背、牛头山林场晋升为国家级森林公园；植树造林30万亩，森林覆盖率达53%；新建沼气4.4万户、节柴灶4.6万座；治理水土流失1196平方公里、小流域96条。西部垃圾场开工建设；10座污水处理厂全部投入使用；主要污染物排放总量继续下降，完成了省定目标。我市基本达到国家卫生城市申报条件，被授予“全国创建文明城市工作先进城市”称号，顺利通过了全国文明城市公共文明指数测评；市交通局、市审计局、黄龙滩水力发电厂被授予“全国精神文明建设工作先进单位”称号；茶店镇、湖北口回族乡、蒋家堰镇被授予“全国创建文明村镇工作先进村镇”称号。市侨联荣获“全国侨联系统先进基层组织”称号。

　　（七）致力创新发展，改革开放取得新突破。各项改革深入推进。企业改制进展顺利，武商集团成功收购人民商场，市属7家企业改制到位，一批久拖未决的疑难问题得到解决。国资监管取得新成效，纳入监管的6家企业经营收入增长19.6%。国有企业产权交易不断规范，交易增值率达到23%。集体林权制度改革有序推进，确权面积和发证面积分别达到92.7%和86.5%。市城投公司体制改革初现成效，净增资本金12亿元，全年融资和偿债近8亿元，土地储备近2000亩。新一轮政府机构改革拉开序幕，改革方案已上报审批。人事制度改革进程加快，事业单位合同管理用人机制初步建立，事业单位绩效工资改革平稳推进。

　　科技创新进一步加快。新增高新技术企业8家，专利申请量增长21.5%，顺利通过国家知识产权试点城市验收。争取国家科技型中小企业创新基金项目20项、省级科技进步奖励项目15项，均居全省前三位；争取省以上科技经费2138万元，创历史记录。“胚胎干细胞”通过省级重点实验室验收、“汽车动力传动和电子控制”通过评审。我市被国家科技部列为中英两国可持续发展对话机制示范试点城市。

　　对外开放和交流合作成效显著。成功举办了2009年南水北调中线核心水源区——湖北十堰·北京推介会、西安·武当旅游推介会、武汉·武当生态文化旅游推介会、十堰·深圳投资说明会，组织经贸考察团对台湾进行了考察交流，邀请了深圳企业投资者联合会来我市考察，开展了“百名老板家乡行”活动，我市与天津河西区、北京丰台区、河北省石家庄市、吉林省四平市以及俄罗斯恩格斯市正式缔结为友好城市，对口援建四川汉源县地震灾区和新疆博乐市工作进展顺利。全市引进项目301个，实际利用外资增长13.6%，利用市外境内资金增长30.4%，外贸出口增长33.6%，外派劳务增长10%。商检、海关、外事、外办等部门积极发挥自身职能，在对外开放和招商引资中作出了积极贡献。

　　（八）统筹城乡发展，社会事业全面进步。教育事业均衡发展。小学、初中入学率分别达到100%和99%；消除中小学危房11万平方米，寄宿制学校基本解决了“吃菜难、如厕难”，75%寄宿制学校解决了“洗澡难”问题。中职招生3.2万人，超出年度目标0.9万人。高考本科上线人数继续达万人以上。十堰职业技术学院完成整体搬迁，郧阳师专一期工程顺利完工，湖北汽车工业学院、郧阳医学院扩建工程正加紧进行，武当山国际武术学院筹建工作有序推进。

　　卫生事业健康发展。新一轮医药卫生体制改革全面启动，城镇居民医疗保险参保率达95%，城区在校生全部参加居民医保；新型农村合作医疗参合率94.9%，提高2.9个百分点。新建和改扩建了15个社区卫生服务机构、200个村卫生室，为20个乡镇卫生院配备了急诊救护车辆，启动了市级医院带县（院）挂乡（院）医疗帮扶活动。有效防治甲流感，成功救治了3例重症甲流患者，实现了患者死亡率、院内感染率、医护人员感染率为零的目标，市卫生局荣获“全国卫生应急先进集体”称号。

　　人口计生事业稳步发展。人口出生率下降0.09个千分点，符合政策生育率提高0.59个百分点，出生婴儿性别比下降0.72个单位值，丹江口市荣获“全国计划生育优质服务先进市”称号。

　　文体事业蓬勃发展。组织开展了庆祝新中国成立60周年和建市40周年庆典活动。《乡试》剧目荣获中国戏剧节剧目奖。史志编纂取得新成果。市艺校晋升为国家级重点中等职业学校。实施了自然村“村村通”广播电视工程，全面启动了数字电视整体转换工作。档案新馆建设已选址。新建和改扩建44个乡镇综合文化站，兴建了623家农家书屋和105个农民健身工程，全民健身活动持续广泛开展。市文体局荣获“全国群众体育先进集体”称号。

　　（九）构建和谐十堰，民生工程取得新进展。就业和社会保障取得明显成效。全市城镇新增就业3.8万人，下岗失业人员再就业1.85万人，我市被确定为首批省级创建创业型城市。社会保险扩面新增8.5万人，城市低保实现应保尽保，农村低保新增3万人，六级以上残疾军人全部纳入城镇职工基本医疗保险。开工建设廉租住房20.4万平方米、4091套，城区开工建设经济适用房18万平方米。改造农村危房6842户、因灾倒房恢复重建2435户。

　　移民安置扎实推进。提前完成了丹江口大坝施工影响区229户、881人的移民搬迁任务，完成了南水北调中线工程791户、3280人的移民外迁试点和6.4万人、178个安置点对接工作，完成了潘口水电站6130户、2.5万人的移民搬迁任务，确保了原迁移民直补资金100%发放到位。

　　社会大局保持稳定。强化社会治安综合治理，深入开展了严打整治、酒后驾车等集中整治行动，确保了全市治安秩序持续平稳，群众安全感和治安满意度分别居全省市州第一位、第二位。强化安全生产监管，全市安全生产事故起数、死亡人数和直接经济损失分别下降4.4%、10.8%和0.27%。组织开展了“信访突击月”和大接访、大排查、大化解以及领导干部“四访促和谐”活动，切实解决了一大批疑难信访案件，信访积案化解率达97%。残联、红十字会、慈善、法律援助等机构热情服务弱势群体，水文、气象、地震等部门积极应灾抗灾，保密、民族宗教、老龄工作得到加强，有力地促进了社会和谐稳定。

　　（十）加强自身建设，政府执行力不断提高。自觉坚持接受市委领导，定期向人大报告工作、向政协通报情况，重大决策征求民主党派、党外人士、人民团体意见，办理人大代表建议168件、政协委员提案356件，满意或基本满意率分别达到100%和98.3%。扎实开展了第二批深入学习实践科学发展观活动，解决了4523个影响和制约科学发展的突出问题。强化政府服务功能，组织开展了“千名干部进百企”、“双联双助”、“重大项目百日攻坚”、“百企联百村”等一系列活动，民主评议了9个市直单位，政府作风和行风得到进一步改进。加强重大投资项目和重点专项资金的审计，提高了资金使用效益，促进了廉政建设。强化社会事务管理，新建了城区12个社区服务站和33个便民服务大厅，电力、邮政、电信等部门服务水平和质量不断提高。深入开展国防教育和“双拥”活动，认真做好征兵、安置、优抚、人防和民兵预备役工作，军政、军民关系进一步密切。着力解决人民群众关心的热点难点问题，市长热线反馈率100%，政府承诺的“十件实事”全面完成。

　　各位代表！回顾过去的一年，我们面对诸多困难和严峻挑战，取得这样的成绩确实不易。这是市委正确领导的结果，是市人大、市政协监督支持的结果，是全市人民深入学习实践科学发展观，不畏艰难、团结奋斗的结果。在此，我代表市政府，向所有参与、支持、关心十堰发展的同志们、朋友们，表示衷心的感谢和崇高的敬意！

　　在总结成绩的同时，也应清醒地看到，我市经济社会发展中一些深层次的矛盾和问题还没有从根本上得到解决，主要是：经济发展总量还不大，与区域性中心城市的地位差距较大；县域经济发展相对滞后，农业产业化、工业化水平不高；投资率低，生产性重大项目少，发展后劲仍然乏力；经济外向度低，对外开放和招商引资还需加大力度；部分低收入群体生活比较困难，移民、环保等任务艰巨、压力加大；有些部门工作创新不足、落实不力，政府自身建设还需进一步加强。对于这些问题，我们将高度重视，积极采取措施，努力加以解决。[FS:PAGE]

　　二、2010年工作思路和主要措施

　　今年是“十一五”和“十二五”规划的承启之年，做好今年的工作，不仅意义重大，而且面临的机遇更多、基础更好、条件更优、加快发展把握更大：国家将继续实施积极的财政政策和适度宽松的货币政策，加大对民生领域和社会事业支持保障力度，有利于我们继续争取项目和资金；国家将启动实施《促进中部地区崛起规划》、《丹江口库区及上游地区经济社会发展规划》、《全国主体功能区规划》，省委省政府继续实施鄂西生态文化旅游圈发展战略，有利于我们争取优惠政策；东风8万辆商用车、三环6万辆和东风实业5万辆专汽、东风渝安20万辆微型车等一批整车扩能项目启动和实施，将推动我市汽车工业新一轮大发展；南水北调中线工程、高速公路、机场、铁路、天然气等一大批重大项目建设，将使我市进入新一轮投资高峰期。尽管国际金融危机还没有结束，影响我市发展的不确定性因素依然存在，但我市进入新一轮加快发展机遇期的基本趋势更加明朗，大发展态势更加看好。我们要备加珍惜来之不易的大好局面，进一步坚定发展信心、创新发展举措，力争经济社会发展好于去年、高于去年、快于去年。

　　2010年经济社会发展的总体要求是：全面贯彻党的十七大和十七届三中、四中全会精神，深入贯彻落实科学发展观，围绕建设区域性中心城市奋斗目标，以项目建设为总抓手，以转变发展方式、调整经济结构为主线，以体制机制创新为动力，着力加快农业产业化进程，着力壮大汽车主导产业，着力发展生态文化旅游业，着力构建立体大交通网络，着力推进“三城联创”，着力推进改革开放和招商引资，着力改善民生促进社会和谐稳定，着力提高政府执行力，努力实现经济社会又好又快发展。

　　2010年全市经济社会发展主要预期目标是：生产总值增长12%；地方一般预算收入增长18%；全社会固定资产投资增长35%；社会消费品零售总额增长20%；利用外资6000万美元；外贸出口1.2亿美元；城镇居民人均可支配收入增长9%；农民人均纯收入增长9%；城镇新增就业3.5万人，登记失业率控制在4.5%以内；居民消费价格总水平、人口自然增长率、万元生产总值综合能耗、主要污染物排放量控制在省定目标以内。经过奋力拼搏，确保生产总值过600亿元、一般预算收入过30亿元、社会消费品零售总额过300亿元，力争工业产值突破1000亿元、固定资产投资突破400亿元。

　　围绕完成预期目标，着力抓好八个方面的工作：

　　（一）着力推进农业产业化进程，带动农村经济新发展。

　　坚持以“一统三分”战略总揽“三农”工作全局，紧紧围绕农民长效增收这个核心，突破性发展农产品加工业，改善农民生产生活条件，促进城乡协调发展，确保农业增加值增长6%、农民人均纯收入新增300元。继续安排1400万元奖励县（市）区和乡镇争先进位。

　　做好“一统三分”战略规划布点工作。因地制宜，突出特色，制定“一统三分”总体规划和专项规划，并把规划具体落实到地域、到产业、到企业、到乡镇、到村组。继续扩大基地规模，确保新增特色基地10万亩，改造升级15万亩，新增畜禽养殖小区100个以上，加快建设农产品加工示范园区、生态农业示范园区、优质高产创建示范园区、现代农业科技示范园区。

　　突破性发展农产品加工业。抢抓省实施“四个一批”农业产业化工程建设机遇，支持龙头企业做大做强，力争龙王垭茶业集团成为国家级龙头企业，茶叶、柑橘、畜牧、烟叶、中药材等每个重点产业至少有1个省级以上的龙头企业、5—10家市级龙头企业，全市农副产品深加工产值突破50亿元。鼓励创办、合办中介组织，新增农民专业合作社60个，提高农民组织化程度。全力打响“武当道茶”品牌，力争产值达到13亿元，增长30%以上。

　　进一步加强农业基础设施建设。继续改造中低产田和低丘岗地，切实抓好54座病险水库加固工程建设，进一步抓好丹江口市脱贫奔小康示范县、7个省级和1个市级新农村示范乡镇建设，全面完成省、市、县三级新农村建设试点村建设任务。扎实推进扶贫开发，减少9万贫困人口，完成5500人的生态移民。启动实施新一轮城区农村扶贫规划。

　　完善农村社会化服务体系。加强对农民工的就业指导、技能培训和信息服务，引导农村劳动力就近转移就业，积极开拓劳务市场，大力发展劳务经济。认真落实扶持返乡创业政策，鼓励农民工返乡创业。切实抓好各类培训项目，提高农民的科技水平和就业能力，力争新增农村劳动力转移3万人以上。继续开展“百企联百村”、“双联双助”活动，提高帮建工作水平。认真落实各类奖补政策，建立农民减负长效机制，坚决防止农民负担反弹。

　　（二）着力壮大汽车主导产业，促进工业经济实现新跨越。

　　坚定工业主导地位不动摇，坚定工业项目建设不放松，坚定结构优化不松懈，坚定服务工业不松劲，推动优势产业集群发展，力求做大规模、调优结构、创新技术、提升品牌，切实增强工业整体实力，力争工业增加值突破300亿元。

　　以重大项目为抓手，切实做优汽车产业。一是全面提升整车生产能力。全力推进10大整车、5大改装车改造工程。继续支持服务东风公司，为“863”振兴计划创造最佳实施环境，力争8万辆商用车扩能项目尽快开工投产、发动机尽量增产增效，确保东风公司传动轴公司整体顺利搬迁。加快推进东风渝安20万辆微型车、三环专汽6万辆和东风实业5万辆专用车、世纪中远5万辆微型车等整车扩能项目，确保整车产量突破50万辆。二是全面提升零部件配套能力。实施100家骨干零部件企业改造工程，加快推进正和6万辆车身扩能、大洋60万套车轮、同创120万套同步器、先锋大型数控压力机、双星东风全钢子午胎等关键零部件重大项目建设，增强发动机、变速箱等关键零部件地产配套能力。利用地方汽车零部件产业的丰富资源，吸引国内外汽车制造商到十堰建立生产基地，拓展零部件市场空间。三是全面提升汽配产品的集聚和辐射能力。加快国际汽车会展中心和国家商用车及零部件检测中心建设，进一步增强中国（十堰）汽配城品牌形象，力争汽配城销售额增长15%以上。

　　以结构调整为主线，加速培育壮大新兴产业。加快发展水电产业，抓好潘口、三里坪等在建的28处75万千瓦水电工程建设，加快孤山、龙背湾等水电站主体工程建设。突破性发展绿色食品工业，推进湖北久康魔芋深加工和功能饮料生产、房县润田矿泉水、农夫山泉天然水项目建设。大力发展生物医药产业，着力推进武当医药工业园建设，支持天圣清大、康迪和开泰激素等医药制造企业开发高端生物医药产品；利用与药都石家庄缔结友好城市的机遇，积极引进知名医药企业，开发本地优质药材资源，提升产业竞争力。抓住国家支持发展低碳经济和循环经济、扶持新兴产业发展的战略机遇，把环保、生物质能源等作为新的支柱产业来培育，力争实现产业、企业、产品的“三突破”，开发生产节能环保的汽配、照明、机电等产品；着力培育生物质能源产业，壮大丹江口油茶、郧西油桐基地规模，支持武汉凯迪等龙头企业发展精深加工。

　　以工业园区为平台，加快产业集群发展。鼓励十堰经济开发区跨域发展，进一步做大做强，确保工业产值增长20%以上。加快开发区和工业园区基础设施建设，加大招商引资力度，吸引大项目和大企业入驻，尽快形成产业集群，培植新的经济增长点。支持县市经济开发区增强产业集聚能力，使之真正成为县域工业发展的主要载体。

　　以创优环境为保障，继续加大服务企业力度。推广“千名干部进百企”经验，探索服务企业的长效机制。全面落实《关于支持十堰城区工业发展的若干政策规定》，市级财政筹集1亿元用于财源建设。继续推进山地整理，加大已开发储备建设用地出让供应力度，按照投资强度、投入产出率、科技含量等控制指标供应土地。建立金融机构和政府部门干部互派交流机制，增进政银沟通交流；鼓励金融机构加大信贷投入力度，确保新增存贷款双超百亿元；引进2-3家埠外金融机构在十堰设点，扩大小额贷款公司试点范围，增强政信担保公司实力，支持符合条件的企业发行债券、票据；助推3-5家企业进入上市辅导期，尽快实现上市。大力推进企业成长工程，鼓励全民创业，扶持中小企业发展，激励“双亿工程”企业做大做强，确保新增规模以上工业企业100家、“双亿工程”企业10家。支持企业争创精品名牌，争取1家以上企业获得长江质量奖、10个产品获得湖北名牌。继续安排1500万元对“双亿工程”和企业创优工作实行奖励。

　　（三）着力发展生态文化旅游业，实现三产发展新突破。

　　以扩大内需为目标，以生态文化旅游业为突破口，不断拓宽三产发展的领域和空间，努力提升服务业档次和水平。

　　打造国际知名生态文化旅游区。充分发挥武当山龙头引领作用，加快旅游项目建设，尽快形成以“三区”、“三线”为重点的旅游精品线路，力争游客人数、旅游收入均增长20%以上。一是在完善旅游规划上求“高”。按照高起点、高标准、高质量的要求，尽快完成旅游发展总体规划修编工作，加快编制特色板块景区控制性详规，努力形成客源互送、线路互推、信息互通、交通互畅的区域旅游发展新格局。二是在旅游重点项目建设上求“快”。加快建设太极湖新区和武当山、丹江口旅游码头、郧阳港，启动市游客集散中心、武当山索道改造、南神道综合开发等重点项目建设，推动旅游业大发展。三是在旅游景区创A上求“多”。加强各类景区创A工作指导，支持赛武当、牛头山、青龙山地质公园等创建4A级景区，鼓励房县创建国家级旅游强县，加速推进旅游业提档升级。四是在旅游商品开发上求“精”。培育和引进一批旅游商品开发企业，积极发掘绿松石、米黄玉、银制品、根雕工艺品、汽车车模、武术及宗教用品等一批具有地方特色的旅游商品，提高旅游消费购物水平。五是在旅游宣传促销上求“新”。抓住举办第四届世界传统武术节的机遇，精心策划营销方案，加强与国内外知名媒体和旅行社合作，全方位、高层次、多角度加强宣传推介，进一步提升旅游业的知名度和市场认可度。同时，依托城郊旅游资源，办好特色节会，培植城郊旅游消费热点。

　　加快区域性现代服务中心建设。做好物流产业发展规划，着力推进火车站商贸经济圈、汽车汽配物流园、汉江物流园三个百亿物流园和九州通医药物流项目建设，提升现代物流业水平。按照规模最大、业态最佳的要求，把人民商场建成一流的区域性商贸中心；做好新建主干道流通网点布局规划，打造精品商贸区，提升商贸流通业规模和层次。支持大型商贸企业、仓储式超市、现代物流配送等新兴业态发展，大力发展农村连锁超市，推进“万村千乡”市场工程和“家电下乡”工程，加快供销合作社改革发展，完善城乡一体的商贸流通体系，促进市场繁荣。

　　（四）着力构建立体大交通网络，开创统筹发展新局面。

　　加快交通建设步伐。把构建内畅外联的现代化立体大交通网络作为打造区域性中心城市的首要任务，全力推进武当山机场立项进程；力争十宜铁路、十三铁路尽快开工；加速推进十房、十白和谷竹三条高速公路建设，开工建设郧十高速公路；确保武白、郧十一级路（郧县段）建成通车，加快郧十一级路（城区段）建设，开工建设丹江口市城区至土关垭、316国道张湾西部复线（即风神大道）一级公路。

　　加强城镇基础设施建设。完成建设大道一期、上海路、北京路南通道、浙江路二期、车站路和十房路口改造工程，开工建设东环路、林荫大道二号线和发展大道，完成48条内街小巷和香港步行街改造，逐步改善城区通行环境。加快推进天然气十堰支干线和城网建设，确保120万千瓦热电联供项目开工建设，启动百二河有水工程，推进上海路北片区综合开发，着手体育公园建设，完善市政环卫基础设施。鼓励和支持县市加快城镇建设步伐，推进城乡一体化进程。

　　（五）着力推进“三城联创”，掀起达标创建新高潮。

　　确保国家卫生城市创建达标。严格对照国家卫生城市创建标准，深入开展城市环境专项整治行动，实施大树进城、裸露山体治理工程，打造迎宾大道，绿化美化亮化净化城市环境，确保顺利实现国家卫生城市创建目标。

　　力争国家环保模范城市创建进入申报程序。认真实施丹江口库区水土保持工程项目，治理水土流失面积717平方公里。继续巩固退耕还林成果，加强天然林保护和长防林、生态公益林建设，完成人工造林30万亩。新建农村户用沼气2万户。推进神定河和泗河流域污水管网建设，完成犟河污水处理厂、西部垃圾处理场建设，启动东部垃圾处理场建设，确保库区水质保持在国家二级标准以上。狠抓重点流域、饮用水源、尾矿库、涉危企业等污染整治，严厉打击环境违法行为。创建全国绿色学校7所和环境优美乡镇、生态示范村、绿色社区各2个以上。

　　夯实全国文明城市创建基础。以养成教育为突破口，提高市民文明素质。加强志愿者队伍建设，提升志愿服务工作水平。创新文明创建活动内容及载体形式，积极争创国家级文明单位、文明社区、文明村镇，确保“省级文明城市”六连冠和“全国创建文明城市工作先进城市”三连冠。加强全民国防教育，做好人民防空和民兵预备役工作，巩固军政军民团结，确保实现全国双拥模范城“六连冠”。

　　全力办好第四届世界传统武术节。精心组织，认真谋划，切实做好筹备工作，力争规模和水平超过上届，努力把第四届世界传统武术节再次办成一流国际性盛会。

　　大力推进城镇化进程。完成城市总体规划修编，尽快完善城乡一体的规划体系，重点加快新建工业园区和新修主干道沿线建设详规，完善配套设施，为工业经济和城镇发展奠定基础。按照“东连北接西优化”的思路，逐步提升中部组团，重点发展东西部组团，加快市县对接进程，拓展城市发展空间。立足区位优势、资源禀赋和产业定位，加强区域性中心镇、边贸口子镇和特色专业镇建设，进一步加快城镇化进程。

　　（六）着力推进改革开放和招商引资，增强经济社会发展新活力。

　　继续深化各项改革。克难攻坚，重点突破，继续抓好应改未改企业的改制重组工作。按照政企分开、政资分开、政事分开原则，全力做好市直机关所属企业和经济实体脱钩改制工作，进一步完善国资监管体系。全面完成集体林权制度改革，逐步建立现代林业产权制度。推广“三村”试点村经验，推进集体资产产权制度改革。抢抓林权、土地流转、集体资产产权等一系列改革机遇，健全完善产权交易市场。继续深化城市建设投融资体制改革，放大城投公司资本金效应。

　　大力推进科技创新。鼓励企业创新达标，力争新增高新技术企业10家、申报国家中小企业创新基金保持全省前三名。积极申报“汽车零部件科技创新示范区”、第三批中小企业成长路线图计划、国家火炬计划和湖北省重大科技计划项目。支持企业与高校、科研院所建立产学研联盟，提高科技成果的转换率。支持高新技术产业、农产品深加工、生物医药企业提高研发水平，坚持资金回收滚存制，确保研发经费持续增长。

　　切实提高招商引资实效。加强招商引资项目的开发、筛选、论证和包装，保证项目库及入库项目数据的实效性和准确性，进一步完善项目库。继续创新方式方法，重点采取产业链招商、园区招商、代理招商和小分队招商等方式，选聘一批市外知名商会、行业协会的主要负责人为政府招商引资顾问。实行分行业、分区域分工负责制，精心组织10大重点招商活动，加大工业新区、汽车产业、生态旅游、农产品加工、郧十高速等重点项目的推介力度，力争以好项目、大项目招好商、招大商。加强督办服务，切实提高合同履约率和资金到位率。建立全员招商责任体系，提高项目前期工作经费，建立考核奖励机制，重奖招商有功单位和人员，充分调动各方面招商的主动性和积极性。

　　进一步扩大外贸出口。抓住国家支持加工贸易转型升级的政策机遇，重点支持整车、轮胎和农产品等特色商品出口。支持市属企业参加国际食品展、美国拉斯维加斯国际汽车展、广交会、世博会、东博会等展会，积极开拓国际市场。

　　（七）着力推进民生工程，营造安定和谐新环境。

　　坚持把解决民生问题作为社会事业发展的重点，着力解决就业、就学、就医、社会保障、社会治安、安全生产等人民群众最关心的利益问题，最大限度地增加和谐因素，全面建设安定和谐新十堰。[FS:PAGE]

　　积极拓展就业岗位，千方百计破解“就业难”。认真开展各类促就业专项活动，积极引导和帮助大中专毕业生、下岗失业人员、返乡农民工就业和再就业，着力援助有劳动能力、有就业愿望的困难人员就业，尽力消除“城镇零就业家庭”和“农村零转移家庭”，确保城镇新增就业3.5万人，帮助下岗失业人员再就业1.6万人。坚持以创业带动就业，积极创建省级创业型城市，帮助2500名城乡劳动者实现创业，带动1万人就业。

　　继续推进扩面提标，提高社会保障水平。重点推进农民工、非公经济组织就业人员、个体工商户、灵活就业人员参保，确保扩面新增6万人。推进农业小三场职工、被辞退民办教师参加养老保险，做好新型农村养老保险试点工作。新增农村低保人口8万人，基本实现应保尽保。进一步加大住房保障工作力度，新增廉租住房补贴10480户，新建廉租住房2000套；力争城区三个经济适用房小区一期工程竣工并交付使用；改造农村危房2.5万户，解决城乡低收入家庭住房困难和住危房问题。加大住房公积金贷款力度，增强中低收入家庭购房能力。改扩建20所农村中心福利院，加快市社会福利服务中心建设，确保市儿童福利院、第二福利院、光荣院和流浪未成年人保护中心建成投入使用。切实保护残疾人权益，提升残疾人事业发展水平。着力做好老龄工作，加快推进以居家养老为基础、社区服务为依托、机构养老为补充的养老服务体系建设。大力发展慈善事业，广泛开展慈善活动。

　　优化教育资源配置，继续解决“上学难”。坚持落实基础教育以县（市）区为主的管理体制，促进义务教育均衡发展。扎实抓好“校安工程”建设，加快推进危房改造步伐，继续改善办学条件，全部解决农村寄宿制学校“吃菜难、如厕难、洗澡难”问题。加快北京路中学建设，缓解城区义务教育资源紧缺矛盾。加快城区职教资源整合，扩大办学规模，提升办学质量，打造品牌院校。加快郧阳师专、湖北汽院、市委党校迁扩建工程建设步伐，发展高等教育和干部培训教育。

　　强化基本医疗保障，努力解决“看病难”和“看病贵”。全面推进医药卫生体制改革，加强新型农村合作医疗制度建设，提高筹资标准和住院报销比例，确保参合率保持在92%以上；切实落实新医保政策，提高待遇水平；初步建立基本药物制度，做好基层医疗机构基本药物配送工作，全面实行基本药品零差价销售；稳步推进公立医院改革试点工作。继续抓好15个社区卫生服务站基础设施和服务能力建设；推进城市医院“挂县带乡连村”帮扶活动，培训500名基层医生，增强基层医疗救治能力。开工建设市突发公共卫生事件应急中心，增强突发公共卫生事件应急处置能力。以稳定低生育水平为目标，全面完成人口和计生省定任务，争创1-2个全国优质服务先进县，努力提高人口素质。

　　积极开展文体活动，丰富人民群众生活。改造30个乡镇综合文化站，支持创作文艺精品，开展丰富多彩的群众性文体活动，做好第三次全国文物普查迎检工作，推进文化事业大繁荣、大发展。全面完成数字电视整体转换工作。全力提高竞技体育水平，力争在省十三届运动会上取得好成绩。继续推进文体设施进社区，积极开展全民健身活动，提高市民健康水平。

　　妥善做好移民安置工作，切实保护移民合法权益。把移民安置作为全市保障民生、维护稳定的头号工程来抓，坚持在稳定中移民、在移民中保稳定。确保完成南水北调中线工程6.4万移民外迁安置和地方水电站1.1万余人的搬迁安置任务。做好大中型水库移民后期规划和扶持工作，确保丹江口、黄龙滩水库六年规划实施顺利通过国家验收，再落实潘口电站8288人移民的后期扶持。

　　深入推进平安十堰建设，全力维护社会和谐稳定。大力推进社会治安防控体系建设，依法严厉打击和严密防范各种违法犯罪活动。加强对重点行业、领域的安全生产监管和专项整治，坚决遏制重大安全生产事故发生。继续开展领导干部“四访促和谐”活动，畅通民情民意诉求渠道，加大矛盾纠纷排查调处力度，注重从源头疏导民怨、化解矛盾。

　　各位代表，民生连着民心，民心汇聚民力，为人民群众办实事是政府应尽的职责。这里，我代表市政府郑重承诺，今年将为全市人民群众继续办好十件实事：一是继续实施扶贫搬迁工程，完成扶贫搬迁11000人。二是新建通村水泥路1000公里，基本实现全市村村通水泥路目标。三是支持20个乡镇建设医疗急救站，提升农村医疗机构应急救治能力。四是扩建市郊9所农村中心福利院，实现郊区农村无基本生活自理能力的五保家庭全部集中供养。五是改扩建市区3所中小学校，缓解农民工子女入学难问题。六是改善市民生活环境，新建山体游园两个、街头游园两个，建设一条景观示范大道。七是市区新建停车场3个，规范恢复停车泊位5000个，新建公厕10座，缓解市民停车难、如厕难问题。八是继续推进饮水安全工程，解决农村16万人饮水不安全问题。九是新建“农家书屋”400家、“农民体育健身工程”100个；市区建设全民健身路径8条、户外公共阅报栏100个。十是加强农村服务体系建设，新建农村村级综合服务社300个。

　　（八）着力提高执行力，树立政府新形象。

　　坚持依法行政，建设法治政府。认真执行重大事项专家论证、公开听证制度，主动听取民主党派、工商联、无党派人士和专家学者的意见和建议，促进政府决策更加科学、民主。认真执行人大及其常委会各项决议，自觉接受人大监督，主动加强与政协的联系，认真办理人大代表议案、建议和政协委员提案。进一步加强电子政务建设，大力推进政府信息公开，充分发挥市长热线、政府部门网站等媒介作用，依法保障人民的知情权、参与权和监督权。完成“五五”普法任务，提高全民法制素质。严格执行执法责任制、执法违法和错案责任追究制，切实解决有法不依、执法不公、执法扰民等群众反映强烈的问题。

　　强化问责监督，建设责任政府。全面完成新一轮政府机构改革，进一步转变职能，理顺职责关系，优化组织结构。严格落实行政问责制，做到执法有保障、有权必有责、用权受监督、侵权须赔偿、违法受追究。加强行政监察，坚决查处不作为、乱作为行为。坚持依法统计，做好第六次全国人口普查工作。

　　提高行政执行力，建设效能政府。加强各级行政服务中心和部门服务窗口建设，实施网上审批、监察，压缩审批时限，提高办事效率。继续深入开展民主评议活动，进一步改进行风。建立强有力的工作推进和目标考评机制，实行重点工作一名领导、一个专班、一抓到底，确保抓出实效。

　　秉承从严治政，建设廉洁政府。深入落实党风廉政建设责任制，狠抓源头治腐，坚决查处腐败案件。严肃财经纪律，加强预算管理，规范公务支出，努力降低行政成本。加大对重点领域、重点部门、重点资金、重点项目的审计监督力度，防止暗箱操作、滋生腐败，树立廉洁政府的好形象。

　　今年，我们还要做好“十二五”规划编制工作，这是事关十堰未来发展的一项重要工作，也是全市人民普遍关注的一件大事。我们将深入调查研究，积极问策纳言，确保高质量、高标准完成规划编制任务，为十堰未来发展绘制既切实可行又鼓舞人心的宏伟蓝图。

　　各位代表，新的一年，政府工作任务繁重而艰巨。我们将在市委的正确领导下，在市人大、政协的监督支持下，充分调动和发挥全市人民的智慧与力量，进一步解放思想、抢抓机遇、迎难而上、创新实干，为圆满完成全年各项目标任务，加快推进区域性中心城市建设而努力奋斗！
